# A Case of Epileptic Convulsions and Hemiplegia, the Consequence of a Contusion on the Head, Cured by the Application of the Trephine: With Observations

**Published:** 1826-02

**Authors:** Andrew Blake

**Affiliations:** Member of the Royal College of Surgeons, and Surgeon to his Majesty's 7th Regiment of Dragoon Guards.


ART. II.—*A Case of Epileptic Convulsions and Hemiplegia, the consequence of a Contusion on the Head, cured by the Application of the Trephine: with Observations.* By ANDREW BLAKE, M.D. Member of the Royal College of Surgeons, and Surgeon to his Majesty's 7th Regiment of Dragoon Guards.

A YOUNG man, of the bilio-sanguineous temperament, a soldier in the 5th regiment of Foot, stationed at Dominica, received, on the 4th of February, 1824, while wrestling with a robust comrade, a blow on the centre of the right parietal bone, from the clenched hand of his opponent; which, however, did not divide the integuments. On the 8th, (four days subsequent to this occurrence,) he came to the regimental hospital, complaining of general symptoms of fever; but did not then mention having received the injury alluded to. The symptoms yielded to the common antiphlogistic treatment in a few days; but headache remained, and gradually became more violent; the pulse at the same time became unusually slow, and the tongue extremely foul. For these complaints he was bled locally and generally, and purged freely: mercury was also administered, and blisters were applied to the head. As soon as the system became affected by the mercury, the pain of the head ceased; and he was discharged from hospital on the 24th of February, in apparent good health.

On the 29th of the same month, however, he returned to that establishment, complaining of pain in the whole of the upper part of the head. The pulse was as slow as sixty in a minute, and came down to fifty-four; the tongue also became thickly loaded, and the pupils were much dilated. A repetition of the treatment which had already been successful was put in practice, but not with a similar result, for the patient did not experience the slightest relief; and, on the 2d of March, after having suffered all day from excruciating headache, (for which he was again bled, &c. &c.) he was seized, at two o'clock P.M. with a fit of epileptic convulsions; on recovering from which he was found to labour under hemiplegia sinistra. The epileptic fits were shortly renewed, and continued, with scarcely any distinct intermissions at first, and none latterly,—the disease then only exhibiting an occasional remission. From the rattling noise, effusion had, to a certain extent, apparently taken place into the trachea; and, from the stertorous breathing, the state of the pulse, and general appearances, a fatal result was hourly expected. Bleeding, the croton oil, purgative and stimulating enemata, were now resorted to; but without any improvement.

Under these distressing circumstances, the application of the trephine to the site of the original injury was, in consultation, deemed the only means, untried, which afforded any chance of success. Accordingly, about seven P.M. I performed the operation in the ordinary way, in presence, and with the assistance, of Dr. Ramsay, surgeon to the Forces. The skull was observed to be exceedingly thick, and but little adhesion seemed to exist between it and the dura mater; but there was no appearance of any fluid collected on or under that membrane, nor was any irregularity of the internal surface of the cranium to be perceived; yet, from the moment the circle of bone was removed, the paroxysms became considerably mitigated, and they ceased altogether in the course of a few hours.

This patient had no return of the epileptic fits, and recovered perfectly the power of his side within less than a month ; the pulse also gradually acquired its natural degree of frequency. It was, however, thought proper to remove him from a tropical climate, as having undergone the operation of the trephine; and, since his return to Europe, I saw him at Chatham in perfect health.

In the 17th volume of the Edinburgh Medical and Surgical Journal, page 322, there is a case analogous to this in its origin, as well as in some of its effects. Dr. Craigie, in a paper he wrote on the Pathological Anatomy of the Human Brain and its Membranes, describes the case briefly thus:—" A man, of about thirty-five years, who had received a slight blow on the head, began, some time after, to labour under pain of the whole syncipital region, and, in the course of a few months, had complete amaurosis of both eyes. Remedial treatment was employed, under eminent surgeons, without much benefit; and, in addition to his other maladies, he soon became epileptic. After suffering, in a hopeless manner, under his complaints for the space of six months, he fell into a comatose state, and died in a few days. On opening the head, it was found that the internal table of the cranium did not adhere with its usual firmness to the dura mater, but dropped off as soon as it was divided all round by the saw. Absorption had rendered the surface of the cranium unequal."

This case, I think, is a fair example of the effects which may arise from a slight blow on the head ; and, from the similarity of its course and leading features to those of my patient, I am led to think that, had the operation not been performed, he must have shared a similar fate.

It may be asked, in what manner did the operation act, as no collection or spicula of bone were discovered ? To this I can only say, that, in addition to its derivative effects, on the principle of the action of the moxa, issues, &c. &c. the determination of blood to the head, in this particular case, may have been relieved on mechanical principles; as it appears, from the experiments instituted by Dr. Kellie, and published in the Transactions of the Medico-Chirurgical Society of Edinburgh, that, in animals on whom the operation of the trepan had been performed, previously to their being bled to death, the brain, on dissection, contained very little blood, (of course, owing to the admission of atmospherical pressure on the brain,) while, in those whose heads were entire, it contained a considerable quantity of that fluid. The brain, also, this gentleman says, continues to fill the cranium in one case, while it subsides within it in the other.

The gradual, but regular, amelioration of the symptoms, in the case here alluded to by me, from the moment the circle of bone was removed, tends, in a great measure, to incline me to attribute the success of the operation to effects referrible to Dr. Kellie's theory. The loose attachment of the dura mater to the cranium, which was observed after the operation, may have also assisted materially in inducing the salutary result, and may also tend to show that Dr. James Johnson's objection* to Dr. Kellie's theory, as contained in page 289 of the Medico-Chirurgical Review for September 1824, is not always applicable to it.

The experiments of Dr. Kellie above alluded to may serve eminently, in the hands of physiologists, to elucidate the theory of certain cases of epilepsy, and likewise to point out principles on which the cure of this obstinate disease may be conducted. I, however, do not pretend at present to do more than simply relate the case as it occurred to me in regimental hospital practice, and endeavour, as far as I can, to explain the principles on which its success may have depended.

*London; November* 1825.

* Dr. Johnson's objection was founded on the intimate adhesion which, in a state of health, exists between the external surface of the dura mater and the cranium, and which the Doctor imagined would counteract atmospherical pressure on the brain.

